# I Am the Master of My Fate

**DOI:** 10.3201/eid2003.AC2003

**Published:** 2014-03

**Affiliations:** Centers for Disease Control and Prevention, Atlanta, Georgia, USA

**Keywords:** Invictus, TB, tuberculosis, Mycobacterium tuberculosis, Nelson Mandela Mural, Nelson Mandela, David Flores, mural, street art, urban art, I am the master of my fate, William Ernest Henley

**Figure:**
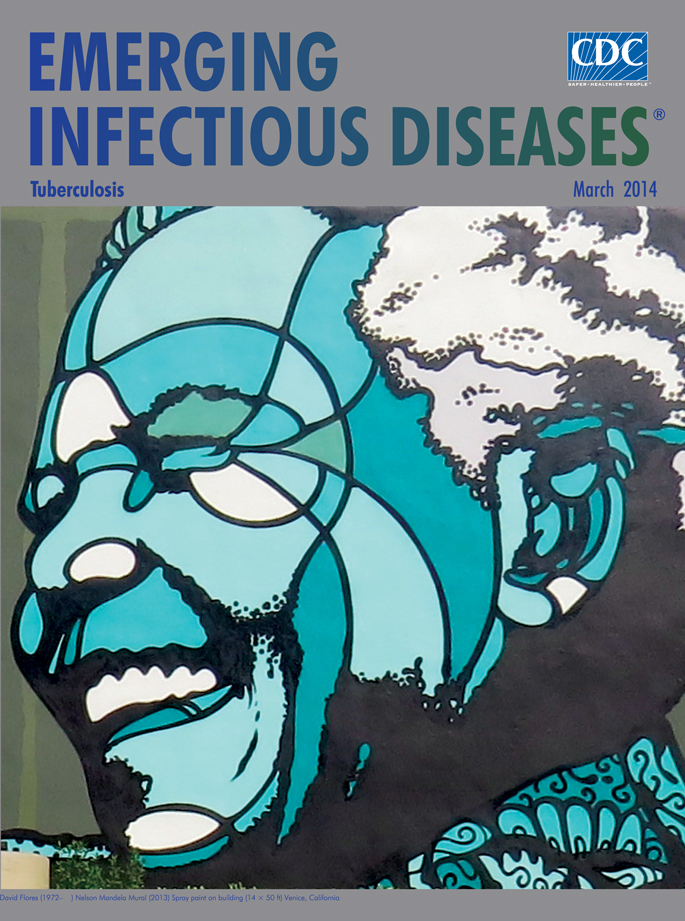
**David Flores (1972– ) Nelson Mandela Mural (2013) Spray paint on building (14 × 50 ft)** Venice, California

“Out of the night that covers me, Black as the pit from pole to pole, I thank whatever gods may be For my unconquerable soul. In the fell clutch of circumstance I have not winced nor cried aloud. Under the bludgeonings of chance My head is bloody, but unbowed.Beyond this place of wrath and tears Looms but the horror of the shade, And yet the menace of the years Finds and shall find me unafraid.  It matters not how strait the gate, How charged with punishments the scroll, I am the master of my fate: I am the captain of my soul.”

Poet William Ernest Henley (1849–1903) penned the words of his immortal *Invictus* (unconquered in Latin) after years of painful tuberculosis (TB) infection of his bones, eventually losing his leg to the disease. Disabled throughout adulthood, and eventually dying of pulmonary TB, Henley nonetheless lived a productive life, thanks in part to his “unconquerable soul.”

Years later, during the incarceration of Nelson Mandela (1918–2013), the words of *Invictus* helped keep hope alive in the South African leader and his fellow prisoners. The same microbe that ravaged Hensley also affected Mandela near the end of his 27 years of imprisonment. When taken to a Cape Town hospital on August 1988, Mandela was unable to speak and had hemoptysis. Fortunately, he recovered after 4 months of treatment—“bloody but unbowed.”

In July 2013, in honor of President Mandela’s 95th birthday, the city of Santa Monica, California, commissioned American artist David Flores to paint a street-side mural. Born in 1972 in California’s Central Valley, Flores studied graphic design in college and swiftly rose to prominence as a commercial and urban artist. He developed an original “stained glass,” mosaic-like style to his portraiture and has become world-renowned for his giant murals of influential figures.

Flores’ complete mural image shows President Mandela freeing a dove that sits in his outstretched hand. (http://davidfloresart.com/blog/mandela/). On a humble, nondescript building, Flores created his postmodern mural with spray paint, using sharp black lines and chromatic variations of a brilliant turquoise, with contrasting patches of bright white. Mandela is depicted wearing one of his signature patterned shirts, and smiling with warmth and determination. The image reminds us that Mandela changed the world with his perseverance and capacity for forgiveness—his smile shining as light through a stained glass window.

In 2004, at the 15th International AIDS Conference, President Mandela spoke about his TB episode in prison. What he said holds true today: “TB remains ignored. Today we are calling on the world to recognize that we can't fight AIDS unless we do much more to fight TB as well.” By 2005, the year his son Makgatho died of AIDS, extensively drug-resistant strains of *Mycobacterium tuberculosis* were beginning to cause lethal HIV-associated hospital outbreaks in parts of South Africa.

TB in prisons is responsible for nearly 10% of the global TB burden. Prisons often offer near-ideal conditions for TB transmission because security concerns obstruct optimal implementation of infection control. The effect of TB in prisons on the incidence of TB in the surrounding community and on the spread of the multidrug-resistant TB (MDR TB) epidemic in the United States has been well described. In more recent years, prisons have also been shown to play an important part in MDR TB transmission in nations of the former Soviet Union and in sub-Saharan Africa. Effective TB control in prisons protects prisoners, staff, visitors and the community at large.

Mandela remained, in the words of Henley, an “unconquerable soul… In the fell clutch of circumstance….” Undefeated by racism, imprisonment, TB, and bitterness, Mandela persevered as the master of his fate. His lasting gift was his power of forgiveness—a gift we remember in his inimitable smile.
